# Rapid and Sensitive Detection of *USP26* Cluster Variants Based on CRISPR/Cas12a

**DOI:** 10.1155/humu/5152235

**Published:** 2026-10-13

**Authors:** Ning Li, Yongkun Huo, Yanan Zhao, Chao Liu, Zhe Zhang, Kaizhao Chen, Longjun Chen, Xiaonan Liu, Ziyang Lin, Ling Sun, Hui Jiang, Xinjie Wang, Wei Li

**Affiliations:** ^1^ Institute of Reproductive Health and Perinatology Guangzhou Women and Children′s Medical Center GMU-GIBH Joint School of Life Sciences Guangzhou Medical University Guangzhou China gzhmu.edu.cn; ^2^ Institute of Brain Research and Rehabilitation South China Normal University Guangzhou China scnu.edu.cn; ^3^ Department of Urology Department of Reproductive Medicine Center Peking University Third Hospital Beijing China puh3.net.cn; ^4^ Department of Urology Institute of Urology Peking University First Hospital Beijing China pkufh.com; ^5^ Genome Analysis Laboratory of the Ministry of Agriculture and Rural Affairs Agricultural Genomics Institute at Shenzhen Chinese Academy of Agricultural Sciences Shenzhen China caas.cn

**Keywords:** CRISPR/Cas12a, detection, infertility, *USP26*

## Abstract

*USP26* gene variants have been reported to be associated with male infertility, such as non‐obstructive azoospermia (NOA), oligozoospermia, and asthenoteratozoospermia, and may be associated with altered sperm sex chromosome segregation patterns. Among the reported variants, *USP26* cluster variants (c.370_371insACA/c.494 T > C/c.1423C > T) represent a frequently reported variant pattern in certain populations. Rapid and convenient detection of *USP26* cluster variants may serve as a powerful tool for male infertility‐associated cohorts. Here, we develop a CRISPR/Cas12a‐based assay to rapid detection *USP26* variants in human samples. Candidate CRISPR RNAs (crRNAs) and RPA primers were screened and optimized, followed by incorporation of restriction enzymes to reduce wild‐type–derived amplification interference. Using controlled artificial DNA mixtures, the assay detected mutant‐template fractions down to 5% under the tested conditions. The complete workflow could be performed within 60 min using basic laboratory equipment. The results of the CRISPR/Cas12a system are consistent with Sanger sequencing in 91 male infertility research samples. Overall, our study establishes a rapid molecular detection method for detecting *USP26* cluster variants and provides an alternative approach for genetic studies of male infertility‐associated cohorts.

## 1. Introduction

Male infertility accounts for approximately half of all global infertility, with a rising prevalence that poses a significant public health burden [1, 2]. Variants in *USP26*, an X‐linked gene encoding a deubiquitinating enzyme, have been reported to be associated with male infertility‐related phenotypes, including non‐obstructive azoospermia (NOA), oligozoospermia, and asthenoteratozoospermia [3–6]. Among those variants, the *USP26* cluster variants (c.370_371insACA/c.494 T > C/c.1423C > T) represent a frequent variant pattern in certain populations [4]. Recent studies have also suggested that men carrying *USP26* cluster variants may show a higher proportion of XY aneuploid sperm than men without these variants, although the potential relationship between these variants and Klinefelter syndrome (KS; 47,XXY) risk requires further investigation [7]. However, the genotype–phenotype relationship of *USP26* variants and their potential contribution to male infertility‐related outcomes or KS susceptibility remain to be further investigated across diverse populations. Thus, rapid and convenient genotyping methods for *USP26* cluster variants may facilitate larger‐scale studies to further characterize their associations with male infertility‐related phenotypes.

Current approaches for genotyping include PCR‐based assays, first‐generation sequencing (FGS; Sanger sequencing), and next‐generation sequencing (NGS). However, these techniques may involve relatively long turnaround times (typically half a day to a full day for Sanger sequencing), high costs, or limited flexibility for rapid detection of specific variants in routine testing settings [8–10]. Therefore, there is a need for detection strategies that combine analytical performance with operational simplicity. Clustered regularly interspaced short palindromic repeats (CRISPR)‐associated systems have recently emerged as powerful molecular detection platforms owing to their rapid and sequence‐specific nucleic acid detection capabilities [11–13]. Although recombinase polymerase amplification (RPA) enables rapid target enrichment under isothermal conditions (37°C–42°C), residual wild‐type (WT) DNA interference may reduce discrimination of variant signals in mutation detection assays [14].

To address WT‐template interference, we previously developed the Excision‐amplification‐synchronous Cas12a‐targeted checkout (EasyCatch) platform, which incorporates restriction digestion into an RPA‐Cas12a workflow to reduce WT‐derived amplification interference [15]. The present study does not introduce a new CRISPR detection principle. Instead, we extended the EasyCatch framework to establish a *USP26* cluster variant‐specific detection assay. The major advances of this study include the de novo design and optimization of variant‐specific CRISPR RNAs (crRNAs) and RPA primers for three *USP26* cluster variants, incorporation of locus‐specific restriction enzyme‐assisted suppression of WT‐derived amplification for two single‐nucleotide variants, establishment of an extraction‐free blood‐processing workflow, and evaluation of the optimized assay using male infertility research samples.

## 2. Methods

### 2.1. Clinical Sample Collection and Processing

Peripheral blood samples were collected from a consecutive cohort of 91 males with infertility at the Center for Reproductive Medicine, Peking University Third Hospital, China. The cohort comprised 17 individuals with asthenospermia and 74 individuals with NOA. No fertile control subjects were included in this study, and no independent external validation cohort was established. The samples were used to evaluate the CRISPR/Cas12a‐based detection workflow for *USP26* cluster variants. Asthenospermia was diagnosed according to the fifth edition World Health Organization (WHO) guidelines. Semen samples were collected by masturbation after 2–7 days of sexual abstinence and analyzed during routine biological examination. Asthenospermia was defined as progressive motility below 32% or total motility (progressive + nonprogressive) below 40%. The inclusion criteria for NOA were (1) no sperm detected in the semen pellets on three different occasions; (2) no evidence of obstruction, inflammation, or injury of the reproductive system or pelvic cavity; and (3) normal karyotype without Y chromosome microdeletions. Individuals without available blood samples for genetic analysis were not included.

All blood samples were processed using a standardized extraction‐free DNA preparation protocol. Briefly, 300 *μ*L of peripheral blood was mixed with 600 *μ*L of red blood cell lysis buffer (1:2, v/v; Solarbio, China) and incubated at room temperature (25°C) for 5 min to lyse erythrocytes while preserving nucleated cells. The sample was subsequently centrifuged at 2320 × g for 1 min to pellet the white blood cells (WBCs). After removal of the supernatant, the WBC pellet was washed with phosphate‐buffered saline (PBS). Genomic DNA (gDNA) was released by incubating the washed WBC pellets with a 20‐*μ*L nucleic acid release reagent (GenDx Biotech, China) at 95°C for 5 min, which disrupted cellular and nuclear membranes and eliminated the need for conventional column‐based extraction. The resulting lysate was used directly as the DNA template for all downstream amplification and detection steps [15].

### 2.2. Plasmid and DNA Fragment Preparation

WT and mutant DNA fragments, spanning lengths of 614 bp (c.370_371insACA), 296 bp (c.494 T > C), and 417 bp (c.1423C > T), were synthesized (TIANYI HUIYUAN, China) and cloned into the pET‐28a vector (GenScript, China). The concentrations of DNA fragments and plasmid were measured using a NanoDrop One spectrophotometer (Thermo Fisher Scientific, United States).

### 2.3. RPA System

RPA was performed using the GenDx ERA Kit (GenDx Biotech Co. Ltd., China). In summary, the 25‐*μ*L RPA reaction mixture contained the following components, as described by Wan et al. [16]: 1.25 *μ*L of the forward/reverse primer (10 nmol/L), 5 *μ*L of the basic buffer, 1 *μ*L of the activator, 10 *μ*L of the reaction buffer, and DNA template. Primer sequences are provided in Table S1. For the c.1423C > T assay, the reverse RPA primer was specifically engineered to introduce the PAM required for Cas12a/crRNA recognition into the RPA amplicon. Because this primer had to simultaneously support efficient RPA amplification, position the introduced PAM appropriately, and maintain compatibility with the corresponding crRNA target sequence, the reverse primer was kept constant, and RPA optimization was performed by screening multiple forward primers in combination with this primer. To improve specificity for the c.494 T > C and c.1423C > T variants, MluCI or BtsCI (New England BioLabs, United States), respectively, was added directly to the RPA reaction mixture at the onset of amplification (1 *μ*L/reaction; ≥10 U), allowing restriction digestion to occur concurrently with RPA amplification and thereby selectively suppressing WT‐derived amplification [15]. The c.1423C > T assay utilized an artificial TCCC PAM introduced through reverse primer engineering to enable Cas12a/crRNA recognition. Reactions were incubated at 37°C for 25 min. Subsequently, RPA products (5 *μ*L) were transferred to the CRISPR/Cas12a detection step. Strict physical separation of pre‐ and postamplification workflows as well as the use of aerosol‐resistant pipette tips and no‐template controls (NCs) were maintained to prevent carryover contamination.

### 2.4. CRISPR/Cas12a Detection and Endpoint Interpretation

The crRNAs, targeting the *USP26* cluster variants (c.370_371insACA/c.494 T > C/c.1423C > T), were synthesized by GenScript (China). The crRNA sequences are provided in Table S2. Cas12a protein was purified according to the protocol described by Creutzburg et al. [17], and the CRISPR/Cas12a reaction was established as described by Wang et al. [18]. The Cas12a reaction mixtures were vortexed and incubated at 37°C for 20 min. During assay development and optimization, fluorescence kinetics were monitored using a fluorescence reader (Ex/Em: 485/520 nm) to evaluate fluorescence signal generation and to compare assay performance under different experimental conditions. After optimization, the established assay was applied for sample detection using a 485‐nm blue‐light transilluminator (Sangon, China) for endpoint fluorescence visualization. Endpoint results were interpreted by visual comparison with the matched negative control. Reactions were classified as positive when a clearly distinguishable green fluorescence signal above the background level of the negative control was observed, whereas reactions showing no visible fluorescence or fluorescence comparable to the negative control were classified as negative. Analytical controls used during assay development and optimization were not considered part of the clinical validation cohort.

### 2.5. Statistical Analysis

All screening and optimization experiments were performed in technical triplicates unless otherwise stated. Statistical analyses were performed using GraphPad Prism 10.0, and data were expressed as mean ± standard error of the mean (SEM). Descriptive statistics were used for assay development experiments and for the analysis of fluorescence signals from clinical samples. Normality and variance homogeneity were confirmed using Shapiro–Wilk and Levene tests, respectively. Differences between groups were analyzed via unpaired two‐tailed Student′s *t*‐tests (two groups) or one‐way ANOVA followed by Tukey′s post hoc test (multiple groups). Significance thresholds were defined as  ^∗^ *p* < 0.05,  ^∗∗^ *p* < 0.01,  ^∗∗∗^ *p* < 0.001, and  ^∗∗∗∗^ *p* < 0.0001 (ns, not significant).

## 3. Results

### 3.1. Establishment of a CRISPR‐Based Detection System for *USP26* Cluster Variants

The *USP26* cluster variants (c.370_371insACA/c.494 T > C/c.1423C > T) have been reported to be associated with NOA, oligozoospermia, and asthenoteratozoospermia, and previous studies have also suggested a potential association between these variants and risk of producing KS offspring [3–6]. We therefore established a target‐specific analytical workflow to support molecular detection of these variants in male infertility research cohorts (Figure 1A). The CRISPR/Cas12a detection workflow consists of two sequential stages: isothermal amplification and Cas12a‐mediated fluorescence reaction. RPA was used to exponentially amplify low‐copy gDNA (1–10 copies) at a constant temperature of 37°C–42°C [14]. Here, we applied RPA to amplify the genomic region spanning the *USP26* cluster variants (c.370_371insACA/c.494 T > C/c.1423C > T), which cleaves a fluorophore‐quencher (FQ) ssDNA reporter upon target recognition. Specifically, upon recognition of the *USP26* cluster variant amplicons, Cas12a exhibited nonspecific ssDNAase activity, resulting in cleavage of the fluorophore‐labeled ssDNA reporter and subsequent fluorescence signals release.

**Figure 1:**
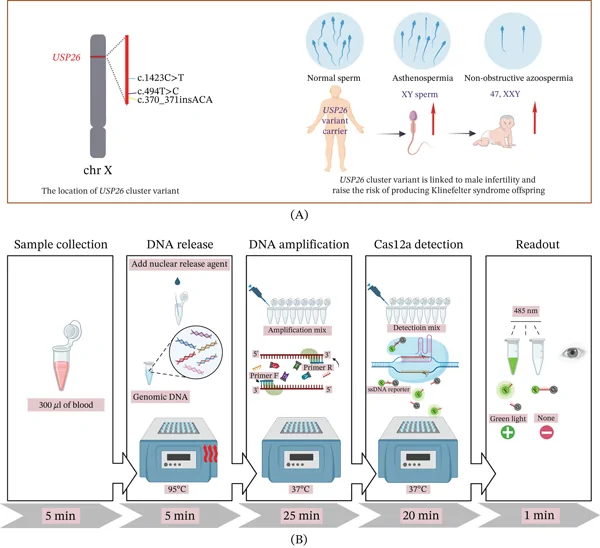
Development of the CRISPR/Cas12a workflow for detecting *USP26* variants associated with male infertility. (A) Genomic locations of *USP26* cluster variants and their association with male infertility and Klinefelter syndrome. (B) Schematic overview of the full workflow from sample collection to visual readout for CRISPR/Cas12a‐based detection of three *USP26* cluster variants. F, forward; R, reverse; ssDNA, single‐stranded DNA.

We established an integrated analytical pipeline from blood processing to readout. The workflow comprised five steps: (1) blood processing (300‐*μ*L blood mixed with 600‐*μ*L red blood cell lysis buffer at room temperature for 5 min); (2) gDNA release (incubation with 20‐*μ*L nucleic acid release reagent at 95°C for 5 min); (3) isothermal amplification (37°C for 25 min); (4) Cas12a reaction (37°C for 20 min); (5) fluorescence readout (Figure 1B). Released gDNA was used directly for RPA, followed by crRNA‐guided Cas12a detection. During assay development and optimization, fluorescence signals were monitored using a fluorescence reader. After optimization, endpoint results were evaluated by visual inspection under a 485‐nm blue‐light transilluminator. Excitation of the green fluorescent signal at 485 nm indicates the presence of *USP26* cluster variants, whereas absence of excitation suggests a variant‐negative result (Figure 1B). The entire procedure, from sample collection to result, takes less than 60 min, and requires basic laboratory equipment.

### 3.2. Design and Optimization of Variant‐Specific crRNAs for *USP26* Cluster Variants

To establish a variant‐specific CRISPR/Cas12a detection system, multiple candidate crRNAs were designed for each *USP26* cluster variant and evaluated for their ability to discriminate mutant from WT targets. Mismatch‐containing crRNAs were additionally designed to reduce potential cross‐reactivity with the corresponding WT sequences (Figure 2A–C). The LbCas12a Ultra enzyme variant was used because of its reported cleavage characteristics and broader PAM compatibility. The performance of the candidate crRNAs was evaluated using 10^10^ copies of mutant or WT DNA fragments, and fluorescence signals were monitored for 30 min to compare target recognition and WT cross‐reactivity (Figure S1A, D, and G).

**Figure 2:**
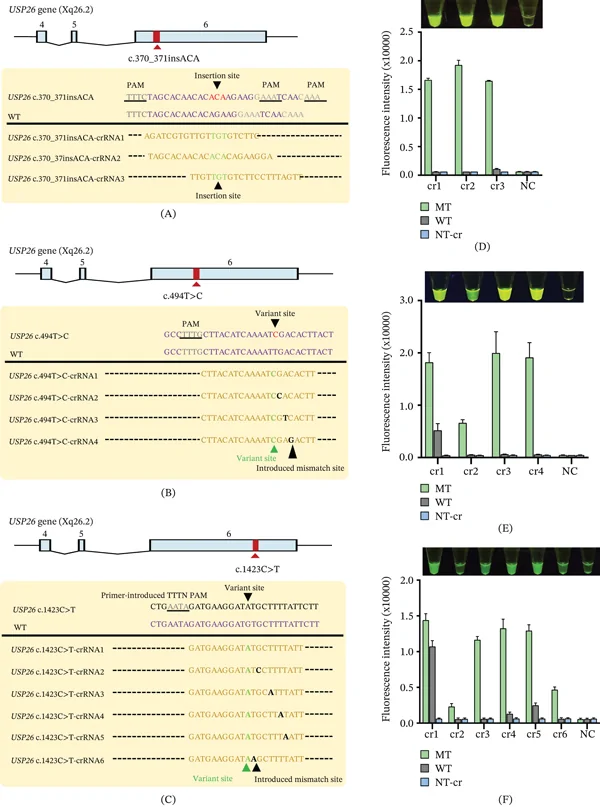
Identification of optimal crRNAs for *USP26* cluster variants. (A) Sequences of three candidate crRNAs targeting c.370_371insACA. PAM sequences are shown in gray and the variant sites in green. (B) Sequences of four candidate crRNAs targeting c.494 T > C. PAM sequences are in gray, introduced mismatches in black, and the variant site in green. (C) Sequences of six candidate crRNAs targeting c.1423C > T. PAM sequences are in gray, mismatches in black, and the variant sites in green. The sequences shown for c.1423 T and c.1423C > T are complementary sequences. (D–F) Visual readouts and fluorescence quantification for detection of 10^10^ copies of wild‐type or mutant DNA fragments using crRNAS targeting (D) c.370_371insACA, (E) c.494 T > C, and (F) c.1423C > T after 20 min. WT, wild‐type DNA template; MT, mutant DNA template; NT‐cr, no‐template with crRNA; NC, no‐template; PAM, protospacer‐adjacent motif; cr, CRISPR RNA.

For the c.370_371insACA site, crRNA2 (cr2) produced the strongest target‐specific fluorescence signal compared with the WT DNA fragment and negative control and was therefore selected for subsequent assay optimization (Figure 2D and Figure S1A, B and C). Similarly, crRNA3 (cr3) showed the best performance among the candidates tested for c.494 T > C and was selected for subsequent experiments (Figure 2E and Figure S1D, E, and F). For c.1423C > T, several candidate crRNAs (cr1, cr2, cr4, and cr5) showed substantial cross‐reactivity with the WT target and were therefore excluded. Among the remaining candidates, cr3 showed better discrimination between the mutant and WT targets than cr6 and was selected for subsequent assay optimization (Figure 2F and Figure S1G, H, and I).

### 3.3. Optimized RPA Primer Screening for Sensitive CRISPR/Cas12a Detection

Following selection of the variant‐specific crRNAs, the RPA primers were optimized to generate amplicons compatible with Cas12a‐mediated detection. We systematically screened RPA primer pairs for the highest amplification efficiency at 37°C. Three forward and three reverse primers flanking the c.370_371insACA locus were designed (Figure 3A). Using 10^5^ copies of the c.370_371insACA DNA as template, we tested the six primer pair combinations (Figure 3B). The forward primer 2 and reverse primer 3 (F2R3) pair for c.370_371insACA was selected as the most efficient based on its superior fluorescence intensity within 20 min of Cas12a reaction at 37°C (Figure 3B and Figure S2A). Similarly, F3R3 was selected for c.494 T > C (Figure 3D,E and Figure S2C). For c.1423C > T, six forward primers were screened in combination with the fixed reverse primer R1. R1 was specifically designed to introduce the PAM required for downstream Cas12a/crRNA recognition. Because the position of the introduced PAM had to remain compatible with the corresponding crRNA target sequence, R1 was fixed rather than subjected to additional reverse‐primer screening. Among the combinations tested, F4R1 produced the strongest Cas12a fluorescence signal and was therefore selected for subsequent experiments (Figure 3G,H and Figure S2E). Because the reverse primer design was constrained by the requirement for PAM introduction and crRNA‐compatible positioning, this screening represented a targeted optimization of forward‐primer performance under a PAM‐constrained design framework rather than an exhaustive bidirectional RPA primer optimization. Additionally, dose‐dependent fluorescence signals were observed across the c.370_371insACA, c.494 T > C, and c.1423C > T sites (10^1^–10^7^ template copies; Figure 3C,F,I). Notably, as few as 10 copies of the c.370_371insACA and c.494 T > C DNA could be successfully detected (Figure 3C,F and Figure S2B and D), and the c.1423C > T assay detected the target at 10^3^ copies (Figure 3I and Figure S2F), which was the lowest copy number tested under these conditions.

**Figure 3:**
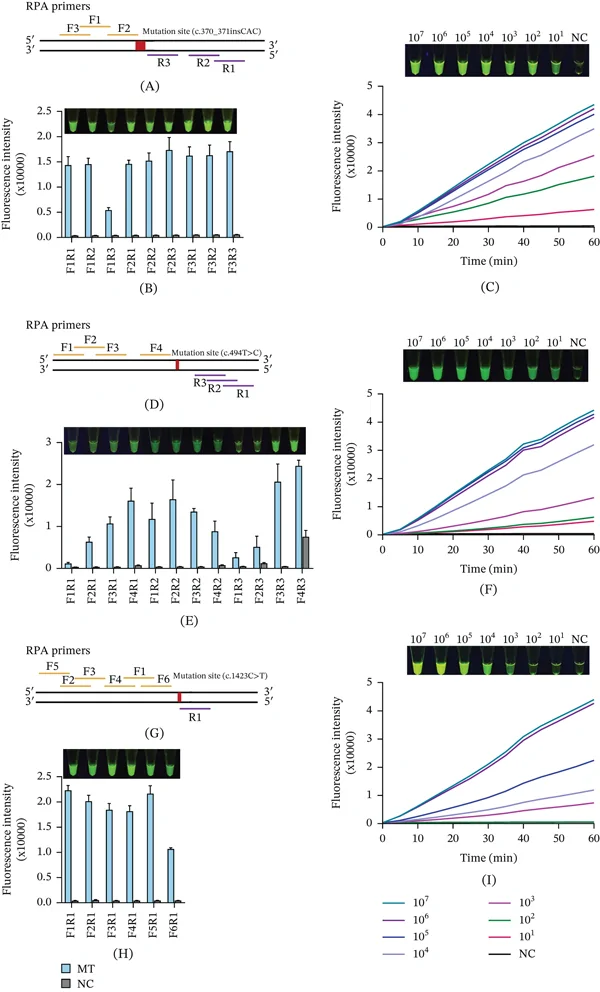
Recombinase polymerase amplification (RPA) primer screening for three *USP26* cluster variants. (A) Genomic positioning of RPA primers flanking the c.370_371insACA site (highlighted in red). (B) Visual readouts (top) and the fluorescence intensity (bottom) for nine primer pairs detecting 10^5^ copies of c.370_371insACA DNA template after 20 min of Cas12a reaction. (C) Time‐course fluorescence analysis of F2R3‐mediated amplification across a gradient of 10^7^–10^1^ copies. (D–F) Corresponding analyses for c.494 T > C, including (D) primer localization, (E) screening results, and (F) time‐course performance of F3R3 primers. (G–I) Corresponding analyses for c.1423C > T, including (G) primer localization, (H) screening results, and (I) time‐course performance of F4R1 primers. MT, mutant DNA template; NC, no‐template; F, forward primer; R, reverse primer.

### 3.4. Specificity Enhancement and Sensitivity Analysis Using Restriction Enzymes

Following optimization of variant‐specific crRNAs and RPA primers, we evaluated the specificity of the CRISPR/Cas12a assays. For the two single‐nucleotide variants (c.494 T > C and c.1423C > T), residual cross‐reactivity with WT templates was observed during the 60‐min monitoring period (Figure S3B, C, E, and G), whereas no detectable cross‐reactivity was observed for the c.370_371insACA locus (Figure 4A–C and Figure S3A and D).

**Figure 4:**
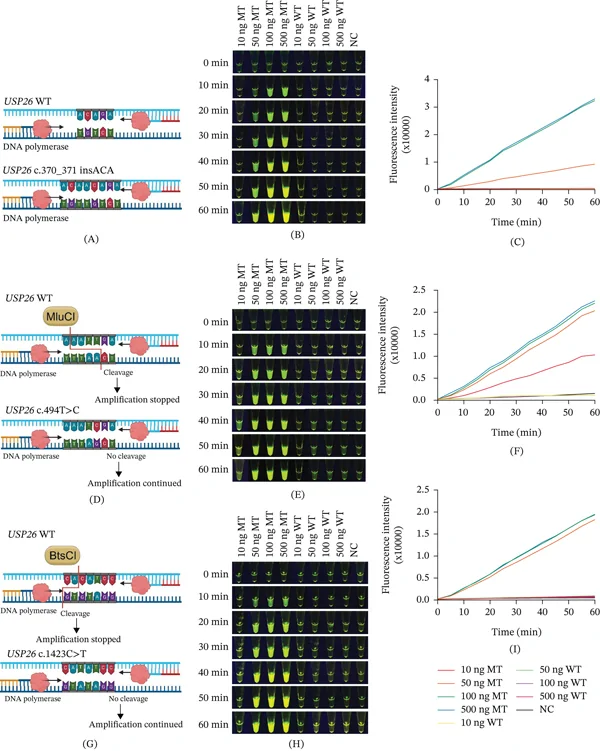
Improvement of CRISPR/Cas12a specificity using restriction enzymes. (A) Schematic of RPA at wild‐type and c.370_371insACA loci without restriction enzyme. (B) Detection results using 500, 100, 50, and 10‐ng gDNA from c.370_371insACA‐positive patients and wild‐type controls without restriction treatment (25‐min RPA, followed by 20‐min Cas12a reaction). (C) Time‐course analysis corresponding to (B). (D) Schematic of RPA with restriction enzyme MluCI at the c.494 T > C locus. The enzyme recognition site (N/AATT) selectively cleaves wild‐type templates and suppresses amplification. (E) Detection results using 1‐*μ*L MluCI (10 U) with 500, 100, 50, and 10‐ng gDNA from mutant and wild‐type samples (25‐min RPA, followed by 20‐min Cas12a reaction). (F) Time‐course analysis corresponding to (E). (G) Schematic of RPA with restriction enzyme BtsCI at the c.1423C > T locus. The enzyme recognition site (CCTAC/NN) suppresses wild‐type amplification. (H) Detection results using 1‐*μ*L BtsCI (10 U) with 500, 100, 50, and 10‐ng gDNA. (I) Time‐course analysis corresponding to (H). WT, wild‐type DNA template; MT, mutant DNA template; NC, no‐template; gDNA, genomic DNA.

To improve discrimination between mutant and WT templates, EasyCatch restriction enzyme‐assisted strategy was incorporated into the RPA‐CRISPR/Cas12a workflow [15]. The restriction enzymes were selected based on the sequence differences between mutant and WT alleles and were added directly to the RPA reaction at the onset of amplification, allowing restriction digestion to occur concurrently with RPA amplification and thereby selectively suppressing WT‐derived amplification. Specifically, MluCI (1 *μ*L, ≥10 U) and BtsCI (1 *μ*L, ≥10 U) were added to the 25‐*μ*L RPA reaction mixture for the c.494 T > C and c.1423C > T assays, respectively. For the c.494 T > C assay, addition of 10 U MluCI effectively suppressed amplification of the WT c.494 T template across the tested DNA‐input range (Figure 4D). In contrast, mutant‐positive c.494 T > C templates remained efficiently amplified and generated strong fluorescence signals with 10–500‐ng gDNA input, without detectable fluorescence signals from WT controls (Figure 4E–F and Figure S3F). Similarly, BtsCI enabled specific detection of c.1423C > T mutant templates across the same DNA‐input range while preventing detectable false‐positive fluorescence signals from WT controls under the tested conditions (Figure 4G–I and Figure S3H). Together, these results demonstrated that restriction enzyme‐assisted amplification improved allele discrimination by reducing WT‐derived interference while maintaining efficient detection of mutant templates. The use of excess enzyme relative to the input gDNA amount was adopted to minimize incomplete digestion and reduce potential false‐positive signals.

Following incorporation of the restriction enzyme‐assisted strategy, visible fluorescence signals were detectable within 20 min using 10–500‐ng gDNA input (Figure 4B,E,H). Collectively, the optimized workflow enabled specific detection of the three *USP26* cluster variants within 60 min with reduced WT cross‐reactivity.

### 3.5. Evaluation of Analytical Detection Capability of the CRISPR/Cas12a Assay for *USP26* Cluster Variants

Analytical detection capability was evaluated by preparing artificial DNA mixtures containing defined mutant‐template fractions of 100%, 50%, 25%, 10%, and 5% by mixing mutant and WT gDNA. This experiment was performed to assess the analytical detection capability of the assay under controlled artificial‐mixture conditions and was not intended to represent the mutant allele fractions encountered in routine germline testing. Across all three loci, a clearly distinguishable fluorescence signal was observed at the lowest mutant‐template fraction tested (5%) under the tested conditions (Figure 5A–I). Because mutant‐template fractions below 5% were not evaluated, the present results do not establish a formal analytical limit of detection. Rather, they demonstrate that the optimized CRISPR/Cas12a assays were able to detect a 5% mutant‐template fraction in the tested artificial DNA mixtures.

**Figure 5:**
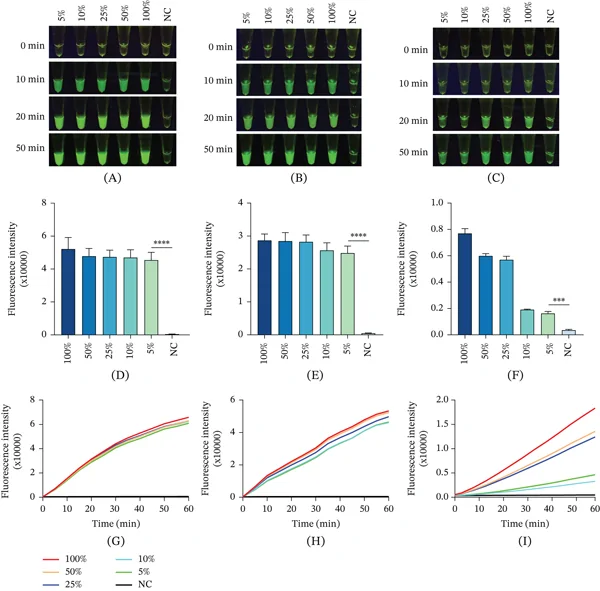
Assessment of the analytical detection capability of the CRISPR/Cas12a assay for *USP26* cluster variants. (A–C) Detection results across mutant‐template fractions (100%, 50%, 25%, 10%, and 5%) for (A) c.370_371insACA, (B) c.494 T > C, and (C) c.1423C > T, showing clearly distinguishable fluorescence signals at the lowest mutant‐template fraction tested (5%). (D–F) Fluorescence intensity measurements at 30 min corresponding to (A–C), respectively. (G–I) Time‐course fluorescence analyses corresponding to (A–C). NC, no‐template.

### 3.6. Evaluation of the CRISPR/Cas12a Assays for Detection of *USP26* Cluster Variants in Male Infertility Samples

To optimize gDNA extraction from blood, we evaluated nucleic acid release time (2, 5, 10, 15, 20, and 25 min) and reagent volumes (20, 50, 100, and 200 *μ*L). A combination of 20‐*μ*L nucleic acid release reagent and 5‐min lysis time provided the best balance between processing time and fluorescence signal intensity (Figure S4A, B, C, and D).

Given the reported association between *USP26* variants and NOA, oligospermia, and asthenoteratozoospermia [19, 20], clinical research samples from patients diagnosed with asthenospermia and NOA were selected for evaluation of the established assay. Using the optimized workflow, we evaluated 91 blood DNA samples from asthenospermia (*n* = 17) and NOA (*n* = 74) patients. No fertile control samples were included in this cohort. The blood processing procedure used for all clinical research samples followed the protocol described in the Methods section (Figure S5). The RPA amplification step (25 min) followed by Cas12a detection (20 min) enabled direct endpoint fluorescence visualization under blue light (Figure 6A).

**Figure 6:**
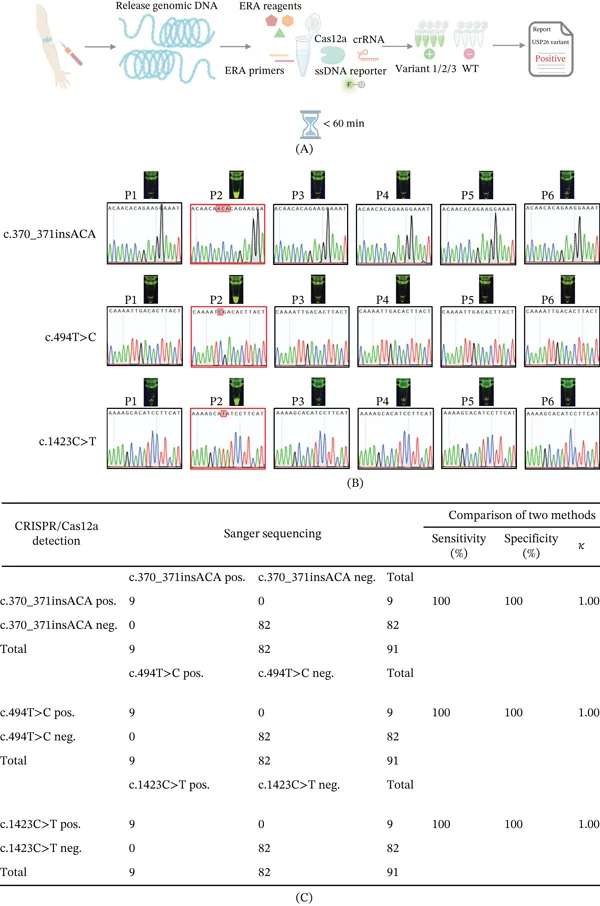
Evaluation of the optimized CRISPR/Cas12a assay using clinical research samples. (A) Schematic of the analytical workflow for clinical research samples. (B) Representative results from six cases comparing CRISPR/Cas12a detection with Sanger sequencing. Variant‐positive samples (c.370_371insACA/c.494 T > C/c.1423C > T) are labeled in red, with mutant bases indicated. (C) Concordance analysis of between CRISPR/Cas12a detection and Sanger sequencing results across 91 clinical gDNA samples, benchmarked against Sanger sequencing. gDNA, genomic DNA.

The established CRISPR/Cas12a assays successfully discriminated samples carrying the three *USP26* cluster variants (c.370_371insACA, c.494 T > C, and c.1423C > T) from variant‐negative samples (Figure 6B and Figure S6, S7, and S8). Among the 91 evaluated samples, 9 individuals carried all three variants (c.370_371insACA, c.494 T > C, and c.1423C > T) (1 out of 17 asthenospermia patients and 8 out of 74 NOA patients), whereas the remaining 82 individuals did not carry these variants. Across the complete cohort of 91 evaluated samples, CRISPR/Cas12a results were concordant with Sanger sequencing for all samples (Figure S6, S7, and S8). Sanger sequencing further confirmed the co‐occurrence of all three variants in the same individuals, supporting their presence as a linked *USP26* variant cluster/haplotype (Figure S6, S7, and S8).

Together, these results demonstrate that the optimized CRISPR/Cas12a workflow enables rapid and reliable detection of the *USP26* cluster variants in the evaluated clinical research samples. The complete assay workflow, including RPA amplification and Cas12a detection, can be completed within approximately 60 min.

## 4. Discussion

*USP26* cluster variants (c.370_371insACA/c.494 T > C/c.1423C > T) have been reported to be associated with male infertility‐related phenotypes, including NOA, oligospermia, and asthenoteratozoospermia [4, 6, 21]. Previous studies have also suggested that these variants may be associated with altered sperm sex chromosome segregation patterns in specific cohorts; however, their contribution to KS (47,XXY) susceptibility remains to be further investigated [7]. Because the three *USP2*6 cluster variants are located within a linked region, conventional genotyping approaches often require multiple PCR amplification steps followed by Sanger sequencing, which can be labor‐intensive and time‐consuming. The present study extends the previously reported EasyCatch framework to a new genetic target, the *USP26* cluster variants, and establishes a target‐specific CRISPR/Cas12a workflow. First, multiple candidate crRNAs were designed and optimized for the three *USP26* cluster variants to achieve variant‐specific Cas12a recognition. Second, a restriction enzyme‐assisted strategy was incorporated to reduce WT‐derived amplification interference and improve discrimination between mutant and WT templates. Third, the optimized assays were evaluated using clinical research samples and demonstrated concordance with Sanger sequencing in the evaluated cohort. This rapid detection workflow can be completed within 60 min and requires basic laboratory equipment. The results obtained using the CRISPR/Cas12a assays showed complete concordance with Sanger sequencing in the 91 evaluated samples, supporting the analytical reliability of the established workflow. Thus, we established a rapid method for detection of *USP26* cluster variants, providing an alternative molecular approach for genetic evaluation in male infertility‐related studies.

Currently, no clinically approved *USP26*‐specific molecular diagnostic test is available. Conventional methods for detecting specific genetic variants, including PCR‐based techniques and sequencing approaches, may require relatively complex workflows, longer turnaround times, or specialized instrumentation. Under our experimental conditions, the estimated reagent cost of the CRISPR/Cas12a assay ($3.75 per sample) was lower than that of qPCR ($5.24 per sample). However, this comparison does not include labor, equipment, and other operational costs. And further evaluation is required to determine its practical advantages over existing laboratory‐based methods. In addition, the EasyCatch‐based workflow enables rapid detection within 60 min using basic laboratory equipment, including a micropipette, heating block, and 485‐nm transilluminator. Therefore, this approach may provide an alternative molecular detection strategy for *USP26* cluster variants in genetic studies and male infertility‐related research cohorts. However, additional clinical studies are required before its application in routine clinical settings.

Clinical studies have suggested that the relationship between *USP26* cluster variants and reproductive phenotypes may be influenced by age and other genetic or environmental factors. For example, oligospermia patients carrying *USP26* cluster variants were reported to be older than fertile men carrying the same variants [7]. However, whether *USP26* cluster variants contribute to progressive age‐related decline in reproductive function remains unclear and requires longitudinal investigation. Mouse studies have further suggested that fertility‐related phenotypes associated with *Usp26* deficiency may depend on age and genetic background [7, 22]. Both pregnancy rates and litter sizes of *Usp26*‐deficient mice were reported to decrease with increasing age, and DBA/2 mice exhibited reduced numbers of round and elongated spermatids compared with C57BL/6 mice [7, 22, 23]. These findings indicate that the biological consequences of *USP26* disruption may involve complex interactions among genetic background, age, and reproductive status. Further studies integrating human genetic data, functional analyses, and longitudinal follow‐up are required to clarify the genotype–phenotype relationship of *USP26* variants.

KS is one of the most common sex chromosome aneuploidies and represents an important genetic cause associated with male infertility [2, 24–26]. Previous studies have suggested a potential relationship between *USP26* cluster variants and altered sperm sex chromosome segregation patterns, including increased proportions of XY aneuploid sperm in specific cohorts [7]. However, whether these variants directly contribute to KS susceptibility remains uncertain, and additional studies involving larger and more diverse populations are required. The CRISPR/Cas12a assay established in this study provides a rapid and convenient approach for detection of *USP26* cluster variants and may facilitate future investigations into the relationship between these variants and reproductive phenotypes. Nevertheless, *USP26* genotyping alone should not currently be considered sufficient for prediction of KS risk or reproductive outcomes.

Several limitations should be acknowledged in the present study. First, the clinical research sample evaluation was performed in 91 male infertility patients without inclusion of fertile controls or an independent validation cohort. Therefore, although the CRISPR/Cas12a assays showed complete concordance with Sanger sequencing in the evaluated samples, the generalizability and clinical utility of this method require further validation in larger and more diverse populations. Second, the genotype–phenotype relationship of *USP26* cluster variants remains incompletely understood, and detection of these variants alone should not be interpreted as a definitive predictor of infertility status, reproductive outcomes, or KS susceptibility.

In summary, this study adapted the established EasyCatch/CRISPR‐Cas12a platform for the specific detection of *USP26* cluster variants, representing an application of this strategy to a previously unexplored genetic target. We designed and validated variant‐specific crRNAs and RPA primers specifically for this cluster and incorporated restriction enzymes (MluCI for c.494 T > C; BtsCI for c.1423C > T) to reduce WT‐derived amplification interference. Evaluation in 91 male infertility research samples demonstrated complete concordance between the CRISPR/Cas12a assays and Sanger sequencing in the evaluated cohort. The established workflow can be completed within 60 min and requires commonly available laboratory equipment for fluorescence visualization. Under the experimental conditions tested, the assay showed reduced reagent costs compared with qPCR; however, further studies are required to evaluate its practical advantages in different laboratory settings. This approach may facilitate molecular genetic studies of *USP26* variants and analysis of male infertility‐associated research cohorts. Future studies involving fertile controls, larger and more diverse populations, blinded testing, independent external validation, and functional investigations will be required to further define the genotype–phenotype relationship of *USP26* cluster variants and clarify their potential clinical significance.

## Author Contributions

All authors contributed to the study conception and design. Ning Li, Yongkun Huo, and Yanan Zhao performed the experimental work of the study, analyzed data, and wrote the manuscript. Kaizhao Chen, Longjun Chen, and Ziyang Lin performed part of the experiments. Wei Li, Hui Jiang, Chao Liu, and Xinjie Wang designed the experiments, supervised the whole project, and revised the manuscript. Zhe Zhang, Xiaonan Liu, Ling Sun, and Hui Jiang provided the experiment samples.

## Funding

This study was supported by the National Key Research and Development Program of China, 10.13039/501100012166, 2022YFC2702600, and the National Natural Science Foundation of China, 10.13039/501100001809, 82522037 32230029.

## Disclosure

All the authors read and approved the final manuscript. Ning Li, Yongkun Huo, Yanan Zhao, Chao Liu, and Zhe Zhang contributed equally to this work.

## Ethics Statement

The study involving human blood samples was approved by the ethics committee of Peking University Third Hospital (IRB00006761‐M2022692) and was conducted in accordance with the Declaration of Helsinki [28]. Written informed consent was obtained from all participants, and all clinical information was anonymized before analysis.

## Conflicts of Interest

The authors declare no conflicts of interest.

## Supporting information


**Supporting Information** Additional supporting information can be found online in the Supporting Information section. Figure S1: Specificity assay of crRNAs targeting the *USP26* cluster variants. Supporting Information 2. Figure S2: RPA primer screening for improved analytical detection of *USP26* cluster variants. Supporting Information 3. Figure S3: EasyCatch‐based improvement of CRISPR/Cas12a assay specificity for USP26 cluster variants. Supporting Information 4. Figure S4: Optimization of gDNA release efficiency. Supporting Information 5. Figure S5: Blood sample processing before CRISPR/Cas12a detection. Supporting Information 6. Figure S6: FGS and CRISPR/Cas12a detection results for the *USP26* c.370_371insACA variant in blood samples from patients with male infertility (P1‐P91). Supporting Information 7. Figure S7: FGS and CRISPR/Cas12a detection results for the *USP26* c.494 T > C variant in blood samples from patients with male infertility (P1‐P91). Supporting Information 8. Figure S8: FGS and CRISPR/Cas12a detection results for the *USP26* c.1423C > T variant in blood samples from patients with male infertility (P1‐P91). Supporting Information 9. Table S1: Primers used for RPA reactions. Supporting Information 10. Table S2: Sequence information for variant crRNAs. Supporting Information 11. Table S3: Comparative cost analysis of qPCR and CRISPR/Cas12a detection approaches for *USP26* cluster variants.

## Data Availability

The data that support the findings of this study are available from the corresponding author upon reasonable request.
