# Supplementary material for: Rapid and Sensitive Detection of USP26 Cluster Variants Based on CRISPR/Cas12a
Source: Hum Mutat. 2026 Oct 13;2026:5152235. doi: 10.1155/humu/5152235 (PMC13662412; doi:10.1155/humu/5152235)
Supplement: Supplementary file 1 [file HUMU-2026-5152235-s001.doc]

**Supplementary Materials**

**Rapid and sensitive detection of *USP26* cluster variants based on CRISPR/Cas12a**

**1. Supplementary Figure S1**

Supplementary Figure S1. Specificity assay of *USP26* cluster variants-crRNAs.

(A) Time-course analysis of c.370_371insACA-crRNA 1-3 to detect variant between WT and mutant DNA fragments for 60 minutes.

(B) Fluorescence heatmap of c.370_371insACA-crRNA-induced Cas12a reactions detecting 10^10^ copies of PCR fragments, Cas12a reactions for 10, 20 and 30 minutes were recorded.

(C) Time-course of optimized c.370_371insACA-crRNA (cr2).

(D) Time-course analysis of c.494T>C-crRNA 1-4 to detect variant between WT and mutant DNA fragment for 60 minutes.

(E) Fluorescence heatmap of c.494T>C-crRNA-induced Cas12a reactions detecting 10^10^ copies of PCR fragments, Cas12a reactions for 10, 20 and 30 minutes were recorded.

(F) Time-course of optimized c.494T>C-crRNA (cr3).

(G) Time-course analysis of c.1423C>T-crRNA 1-6 to detect variant between WT and mutant DNA fragment for 60 minutes.

(H) Fluorescence heatmap of c.1423C>T-crRNA-induced Cas12a reactions detecting 10^10^ copies of PCR fragments, Cas12a reactions for 10, 20 and 30 minutes were recorded.

(I) Time-course of optimized c.1423C>T-crRNA (cr3).

WT, wild-type DNA template; MT, mutant DNA template; NC, no-template; cr, CRISPR RNA.

**2. Supplementary Figure S2**

Supplementary Figure S2. RPA primer screening for sensitive *USP26* cluster variants detection.

(A) The result of the endpoint fluorescence visualization of c.370_371insACA primer pairs mediated RPA using 10^5^ copies of c.370_371insACA DNA templates for 60 minutes.

(B) The result of the endpoint fluorescence visualization of c.370_371insACA F2R3 mediated RPA using 10^7^ to 10^1^ gradient copies of c.370_371insACA DNA templates for 60 minutes.

(C) The result of the endpoint fluorescence visualization of c.494T>C primer pairs mediated RPA using10^5^ copies of c.494T>C DNA templates for 60 minutes.

(D) The result of the endpoint fluorescence visualization of c.494T>C F3R3 mediated RPA using 10^7^ to 10^1^ gradient copies of c.494T>C DNA templates for 60 minutes.

(E) The result of the endpoint fluorescence visualization c.1423C>T primer pairs mediated RPA using 1×10^5^ copies of c.1423C>T DNA templates for 60 minutes.

(F) The result of the endpoint fluorescence visualizationc.1423C>T F4R1 mediated RPA using 10^7^ to 10^1^ gradient copies of c.1423C>T DNA templates for 60 minutes.

NC, no-template; F, forward primer; R, reverse primer.

**3. Supplementary Figure S3**

Supplementary Figure S3. EasyCatch for improving the specificity of the CRISPR/Cas12a detection system for *USP26* cluster variants.

(A-C) Comparison of fluorescence intensity of different amount of mutant DNA (500, 100, 50, 10 ng) of c.370_371insACA (A), c.494T>C (B), and c.1423C>T (C) after 60 min reaction between mutant and WT.

(D, E, G). Time-course analysis of different variant copies (10^10^, 10^11^, 10^12^) of c.370_371insACA (D), c.494T>C (E), and c.1423C>T (G) in 60 minutes without restriction enzymes.

(F, H). Time-course analysis of different mutant copies (10^10^, 10^11^, 10^12^) of c.494T>C (F), and c.1423C>T (H) in 60 minutes with restriction enzymes.

WT, wild-type DNA template; MT, mutant DNA template; NC, no-template.

**4. Supplementary Figure S4**

Supplementary Figure S4. Optimization of gDNA release efficiency.

(A) Time-course analysis of different volumes of nucleic acid release reagent (20, 50, 100, 200 μL) of normal human blood in 60 minutes.

(B) Time-course analysis of different nucleic acid release times (2, 5, 10, 15, 20, 25 min) of normal human blood in 60 minutes.

(C) Comparison of fluorescence intensity of different volumes of nucleic acid release reagent (20, 50, 100, 200 μL) of normal human blood after 60 min reaction.

(D) Comparison of fluorescence intensity of different nucleic acid release times (2, 5, 10, 15, 20, 25 min) of normal human blood after 60 min reaction.

MT, mutant DNA template; NC, no-template.

**5. Supplementary Figure S5**

Supplementary Figure S5. Blood sample processing before CRISPR/Cas12a detection. Briefly, peripheral blood drawn from a patient with *USP26* cluster variant was equally divided into 300 µl/sample, then mixed with 600 µl red blood cell (RBC) lysis buffer. Then white blood cells (WBC) were collected by centrifugal precipitation for 1 min, processed by nucleic acid release reagent and detected using optimized CRISPR/Cas12a detection system.

**6. Supplementary Figure S6**

**A**

Supplementary Figure S6. FGS and CRISPR/Cas12a detection results of the *USP26* c.370_371insACA variant of blood samples from patients with male infertility. Positive agreement was 100% (9/9; 95% CI, 66.4%-100%), negative agreement was 100% (82/82; 95% CI, 95.6%-100%), and overall agreement was 100% (91/91; 95% CI, 96.0%-100%). (A) Samples P1-P24. (B) Samples P25-P48. (C) Samples P49-P72. (D) Samples P73-P91. FGS results were shown in peaks and variant bases were marked in red. Both time-course and naked-eye result of CRISPR/Cas12a detection are shown below.

**Supplementary Figure S6**

**B**

**Supplementary Figure S6**

**C**

**Supplementary Figure S6**

**D**

**7. Supplementary Figure S7**

**A**

Supplementary Figure S7. FGS and CRISPR/Cas12a detection results of the *USP26* c.494T>C variant of blood samples from patients with male infertility. Positive agreement was 100% (9/9; 95% CI, 66.4%-100%), negative agreement was 100% (82/82; 95% CI, 95.6%-100%), and overall agreement was 100% (91/91; 95% CI, 96.0%-100%). (A) Samples P1-P24. (B) Samples P25-P48. (C) Samples P49-P72. (D) Samples P73-P91. FGS results were shown in peaks and variant bases were marked in red. Both time-course and naked-eye result of CRISPR/Cas12a detection were shown below.

**Supplementary Figure S7**

**B**

**Supplementary Figure S7**

**C**

**Supplementary Figure S7**

**D**

**8. Supplementary Figure S8**

**A**

Supplementary Figure S8. FGS and CRISPR/Cas12a detection results of the *USP26* c.1423C>T variant of blood samples from patients with male infertility. Positive agreement was 100% (9/9; 95% CI, 66.4%-100%), negative agreement was 100% (82/82; 95% CI, 95.6%-100%), and overall agreement was 100% (91/91; 95% CI, 96.0%-100%). (A) Samples P1-P24. (B) Samples P25-P48. (C) Samples P49-P72. (D) Samples P73-P91. FGS results were shown in peaks and variant bases were marked in red. Both time-course and naked-eye result of CRISPR/Cas12a detection were shown below.

**Supplementary Figure S8**

**B**

**Supplementary Figure S8**

**C**

**Supplementary Figure S8**

**D**

**9. Supplementary Table S1. Primers for RPA reactions.**

| **Name** | **Sequence** |
| --- | --- |
| **c.370_371insACA-F1** | 5'-gaggttcagccacctgtgagacctggtaag-3' |
| **c.370_371insACA-F2** | 5'-acctggtaagggtgggagtgtcttttctag-3' |
| **c.370_37 insACA-F3** | 5'-caaaacgaggttcagccacctgtgagacct-3' |
| **c.370_371insACA-R1** | 5'-tgtaagcaaaggcatcctctgaaggacacc-3' |
| **c.370_371insACA-R2** | 5'-ggacacctgtcccacttccttttgctatct-3' |
| **c.370_371insACA-R3** | 5'-agcaaaggcatcctctgaaggacacctgtc-3' |
| **c.494T>C-F1** | 5'-ttcttggacagagttcatcaaaacgaggttcagc-3' |
| **c.494T>C-F2** | 5'-agttcatcaaaacgaggttcagccacctgtg-3' |
| **c.494T>C-F3** | 5'-agccacctgtgagacctggtaagggtgggag-3' |
| **c.494T>C-F4** | 5'-aatcttttgagatagcaaaaggaagtgggac-3' |
| **c.494T>C-R1** | 5'-cctcttcttgtgctgattttctgataactc-3' |
| **c.494T>C-R2** | 5'-agctagatgagagcattcttttcctcttcttg-3' |
| **c.494T>C-R3** | 5'-cttgtgctgattttctgataactctccgca-3' |
| **c.1423C>T-F1** | 5'-attctcaagacagaactgaataattacctctc-3' |
| **c.1423C>T-F2** | 5'-ctgtcattactaattttgagttagagttgttgc-3' |
| **c.1423C>T-F3** | 5'-agttagagttgttgcactccattgcttgtaaagc-3' |
| **c.1423C>T-F4** | 5'-attgcttgtaaagcttgtggtcaggttattctc-3' |
| **c.1423C>T-F5** | 5'-ttttcttgccctgtcattactaattttgag-3' |
| **c.1423C>T-F6** | 5'-aaagatcaaaagtagactgTTtagatgaag-3' |
| **c.1423C>T-R1** | 5'-tgaataattacctctccatcaaccttcccc-3' |

**F, forward primer; R, reverse primer.**

**10. Supplementary Table S2. Sequence information for variant crRNAs.**

| **Name** | **Sequence** |
| --- | --- |
| **c.370_371insACA-cr1** | cttctgTGTtgttgtgctaga |
| **c.370_371insACA-cr2** | tagcacaacaACAcagaagga |
| **c.370_371insACA-cr3** | ttgatttccttctgTGTtgtt |
| **c.494T>C-cr1** | cttacatcaaaatCgacactt |
| **c.494T>C-cr2** | cttacatcaaaatCCacactt |
| **c.494T>C-cr3** | cttacatcaaaatCgTcactt |
| **c.494T>C-cr4** | cttacatcaaaatCgaGactt |
| **c.1423C>T-cr1** | gatgaaggatAtgcttttatt |
| **c.1423C>T-cr2** | gatgaaggatgtCcttttatt |
| **c.1423C>T-cr3** | gatgaaggatgtgcAtttatt |
| **c.1423C>T-cr4** | gatgaaggatgtgcttAtatt |
| **c.1423C>T-cr5** | gatgaaggatgtgctttAatt |
| **c.1423C>T-cr6** | gatgaaggatgAgcttttatt |

cr, CRISPR RNA.

**Supplementary Table S3. Comparative cost analysis of qPCR and CRISPR/Cas12a detection approaches for *USP26* variants.**

| ****Component**** | ****Detection methods**** | | |
| --- | --- | --- | --- |
|  | ****qPCR (Probe-based)**** | ****CRISPR/Cas12a Detection****  (Clinical/Economy Type for Primary/Routine Screening) | ****CRISPR/Cas12a Detection****  (Laboratory Standard Type for Research/High-Accuracy Detection) |
| ****Core Enzyme**** | Takara Probe  qPCR Mix $1.25 | GenScript LbCas12a Nuclease  $0.77 | GenScript LbCas12a Nuclease $0.77 |
| ****Amplification System**** | NEB Luna Probe Master Mix $2.99 | GenDx RPA Kit (clinical procurement price) $1.68 | GenDx RPA Kit (laboratory Conventional price)  $4.20 |
| ****Primers**** | Specific primers $0.28 | Specific primers $0.28 | Specific primers $0.28 |
| ****Probe/Reporting System**** | Biosyn Custom  TaqMan Probe: $0.70 | 23nt ssDNA-FAM/BHQ  (GENEMIZ Biotech) $0.49 | 23nt ssDNA-FAM/BHQ  (GENEMIZ Biotech) $0.49 |
| ****Guide RNA**** | Not Applicable | GenScript 23nt crRNA  $0.49 | GenScript 23nt crRNA  $0.49 |
| ****Buffer/Auxiliary Reagents**** | Included (in qPCR Mix) $0.00 | NEB Buffer 3.1 $0.022 | NEB Buffer 3.1 $0.022 |
| ****Lysis Buffer**** | GenDx Nucleic Acid Release Reagent (NR201) $0.018 | GenDx Nucleic Acid Release Reagent (NR201) $0.018 | GenDx Nucleic Acid Release Reagent (NR201) $0.018 |
| ****Total Cost per Test**** | $5.24 | $3.75 | $6.27 |
